# How font size affects judgments of learning: Simultaneous mediating effect of item-specific beliefs about fluency and moderating effect of beliefs about font size and memory

**DOI:** 10.1371/journal.pone.0200888

**Published:** 2018-07-20

**Authors:** Ningxin Su, Tongtong Li, Jun Zheng, Xiao Hu, Tian Fan, Liang Luo

**Affiliations:** 1 Collaborative Innovation Center of Assessment Toward Basic Education Quality, Beijing Normal University, Beijing, China; 2 State Key Laboratory of Cognitive Neuroscience and Learning, Beijing Normal University, Beijing, China; 3 Mental Health Education and Counseling Center, Hefei University of Technology, HeFei, China; 4 Institute of Developmental Psychology, Beijing Normal University, Beijing, China; Southwest University, CHINA

## Abstract

Numerous studies have provided experience-based or theory-based frameworks for the basis of judgment of learning (JOL). However, few studies have directly measured processing experience and beliefs related to the same cue in one experiment and examined their joint contribution to JOLs. The present study focused on font-size effects and aimed to examine the simultaneous contribution of processing fluency and beliefs to the effect of font size on JOLs. We directly measured processing fluency via self-paced study time. We also directly measured participants’ beliefs via two approaches: pre-study global differentiated predictions (GPREDs) as an indicator of preexisting beliefs about font size and memory and ease of learning judgments (EORs) as online generated item-specific beliefs about fluency. In Experiment 1, EORs partially mediated the font-size effect, whereas self-paced study time did not. In Experiments 2a and 2b, EORs mediated the font-size effect; at the same time, beliefs about font size and memory moderated the font-size effect. In summary, the present study demonstrates a major role of beliefs underlying the font-size effect.

## Introduction

Judgment of learning (JOL) [1] is a main measure of learners’ metamemory monitoring of the ongoing learning process wherein participants are asked to predict their future recall performance for items studied. Researchers have long discussed how people make JOLs and have proposed frameworks for the basis of JOLs [2–5]. The cue-utilization view [4] derives two types of frameworks for the basis of JOLs, experience-based accounts and theory-based accounts [6, 7], which are widely accepted by researchers. Experience-based frameworks suggest that people make JOLs based on their experiences or feelings (e.g., the experienced ease of processing or processing fluency) associated with the studying process. Theory-based frameworks suggest that people possess beliefs about how manipulated cues influence memory and then make JOLs accordingly. Numerous studies have been conducted to directly measure processing experience and/or beliefs to demonstrate how processing experience and/or beliefs form the basis of JOLs [8–12]. We reviewed these studies and constructed a table (Table 1) to organize the findings.

**Table 1:** Studies supporting processing experience and/or beliefs as the basis for JOLs. **Note**. ST = self-paced study time, EORs = ease of reading judgments, TTA = numbers of trials to acquisition, GPREDs = global differentiated predictions.

	Manipulation	Direct measures of processing experience	Direct measures of beliefs	Basis of JOLs
Hertzog, Dunlosky, Robinson and Kidder [13]	Interactive image	Generating latency	No	Encoding fluency
Koriat and Ma’ayan [14]				
Experiment 1	Pre-JOL recall	ST, retrieval success & latency	No	Encoding fluency & retrieval fluency
Experiment 2	Pre-JOL recall	Retrieval success & latency	No	
Robinson, Hertzog and Dunlosky [15]				
Experiment 1	Interactive image	Generating success & latency	No	Encoding fluency
Experiment 2	Interactive image	Generating success & latency, perceived generating latency	No	
Undorf and Erdfelder [16]	No	ST	No	Encoding fluency
Undorf and Erdfelder [17]	No	ST	No	Encoding fluency
Beskin and Mulligan [18]	Visual interference	Identification accuracy	No	Perceptual fluency
Beskin and Mulligan [19]	Auditory generation	Identification accuracy or latency	No	Perceptual fluency
Undorf and Erdfelder [20]				
Experiment 1	Relatedness	TTA	No	Processing fluency
Experiment 2	Relatedness	ST	No	Processing fluency
Susser and Mulligan [21]				
Experiments 1 & 2	Hand dominance	Writing time	No	Motoric fluency
Experiment 3	Hand dominance	No	GPREDs	
Undorf, Zimdahl and Bernstein [8]				
Experiments 1–3	Clarification speed	Identification time	No	Perceptual fluency
Experiment 4	Clarification speed	Identification time	Observer’s JOLs	
Yang, Huang, and Shanks [22]				
Experiments 1& 2	Font size	Identification speed	No	Perceptual fluency
Experiment 3	Font size	Identification speed	Observation JOLs	
Matvey, Dunlosky and Guttentag [23]	Target generation	Generating latency	Observer’s JOLs	Belief about fluency and memory
Mueller, Tauber and Dunlosky [12]				
An initial evaluation survey	Relatedness	No	GPREDs	Belief about relatedness and memory
Experiment 1	Relatedness	No	Pre-study JOLs	
Experiment 3	Relatedness	Lexical decision time	No	
Mueller, Dunlosky, Tauber and Rhodes [11]				
Experiment 1	Font size	Lexical decision time	No	Belief about font size and memory
Experiment 2	Font size	ST	No	
Experiments 3a & 3b	Font size	No	GPREDs	
Experiment 4	Font size	No	Pre-study JOLs	
Hu, Li, Zheng, Su, Liu and Luo [24]				
Experiment 1	Font size	No	Observer’s JOLs	Belief about font size and memory
Experiment 2	Font size	No	GPREDs	
Li, Hu, Zheng, Su, Liu and Luo [25], Experiment 4	Visual mental imagery size	No	GPREDs	Belief about visual mental imagery size and memory
Li, Jia, Li and Li [26]				
Experiment 2a	Animacy	ST	No	Belief about animacy and memory
Experiment 3	Animacy	No	GPREDs	
Jia, Li, Li, Zhang, Cao, Cao et al. [27]				
Experiment 2a	Word frequency	ST	No	Belief about word frequency and memory
Experiment 3a	Word frequency	No	GPREDs	
Experiment 3b	Word frequency	No	Pre-study JOLs	
Susser, Jin and Mulligan [10]	Identity priming	Naming latency	No	Belief
Mueller, Dunlosky and Tauber [5]				
Experiment 1	Identical word pairs	ST	No	Belief about identity and memory
Experiment 3	Identical word pairs	No	Pre-study JOLs	
Experiment 4	Identical word pairs	No	GPREDs	
Witherby and Tauber [28]				
Experiment 1	Concreteness	No	GPREDs	Belief about concreteness and memory
Experiments 2 & 3	Concreteness	No	Pre-study JOLs	
Experiment 4	Concreteness	Lexical decision time	No	
Experiment 5	Concreteness	ST	No	
Experiment 6	Concreteness	TTA	No	
Experiment 7	Concreteness	Image latency	No	
Frank and Kuhlmann [9]	Volume	No	Belief: GPREDs; experience: no	Experience & belief about volume and memory

As Table 1 shows, although a sizable number of studies have demonstrated that manipulated cues influence JOLs based on processing experience or beliefs about memory, few studies have examined whether and how one cue can impact JOLs based on processing experience and beliefs simultaneously during the same study phase. One exception is Frank and Kuhlmann [9], who explored the contribution of experience and/or beliefs to the volume effect [29], whereby people give higher JOLs to louder words. They measured participants’ preexisting beliefs about volume and memory via global differentiated predictions (GPREDs) [30]. GPREDs of quiet words were subtracted from GPREDs of loud words as “beliefs”. Then, “beliefs” and volume were entered into a mixed model predicting JOLs (level 1: items, level 2: participants). The results suggested that after controlling for beliefs, volume still significantly affected JOLs, which indicated that something other than beliefs (i.e., processing experience) influenced JOLs. Moreover, there was a significant interaction between volume and beliefs: the stronger the belief that louder words would be recalled better, the more JOLs would be given to louder words than to quiet words, suggesting that beliefs about volume and memory may moderate the volume effect.

Although Frank and Kuhlmann [9] were the first to demonstrate the simultaneous contribution of processing experience and beliefs to JOLs, their study can be improved. First, because the study did not directly measure processing experience, it could not describe the type of processing experience observed. As the authors stated in the discussion, “it remains an open question as to whether these experience-based cues contributing to the volume effect involve processing fluency, embodied cognition, or something else”. Second, although their study was the first to link preexisting beliefs to item-level JOLs, the possible contribution of online generated beliefs to JOLs was omitted [5, 31]. Moreover, whether the results are universal and underlie the effects of other cues on JOLs must be further examined. By exploring the effect of font size rather than volume on JOLs, the present study aimed to directly measure processing experience and beliefs and to further examine whether and how these factors simultaneously influence JOLs.

Font size was chosen in the present study because font size is a cue similar to volume, which is also a type of perceptual characteristic. In addition, similar to volume, font size can significantly affect people’s JOLs (people give higher JOLs to large words than to small words) without influencing actual recall performance, which is called the font-size effect [32]. More importantly, research has shown the contribution of processing experience and beliefs about font size and memory to the font-size effect [32, 11, 24]. A study conducted by Hu et al. [24] may provide evidence supporting the possible co-occurring contribution of processing experience and beliefs about font size and memory to the font-size effect. These authors found that people’s beliefs about font size and memory (measured by differences in GPREDs of large and small words) could explain approximately 20% of the difference in JOLs, with the remaining variation likely explained by processing experience. We reanalyzed the data of Hu et al. utilizing the mixed model conducted by Frank and Kuhlmann [9]. We found that the main effect of font size and the interaction between font size and beliefs about font size and memory were both significant (for basic descriptive statistics and the results of the multilevel model, see Table A and Table B in S1 Table), a pattern similar to that found in Frank and Kulhmann [9], revealing the simultaneous contribution of processing experience and beliefs about font size and memory to the font-size effect. However, the same question remained: what is the type of processing experience? Do processing experience and beliefs about font size and memory contribute to the font-size effect simultaneously during the same study phase, and if so, how? The present study aimed to answer these questions.

Processing fluency is the most widely explored type of processing experience [33] and refers to people’s subjective experience of the ease with which they process information. According to Table 1, processing fluency contributes to the effects of various cues on JOLs. Some studies have proved that processing fluency is a mediator [8, 20]. Processing fluency has also been assumed to mediate the effect of font size on JOLs [32]; large-font-size words produced higher processing fluency than smaller words, which in turn led to higher JOLs. Research has shown that when fluency produced by font size was disrupted by words with aLtErNaTeLy capitalized letters, JOLs were no longer affected by font size, demonstrating that processing fluency might underlie the font-size effect [32]. Nevertheless, when processing fluency was directly measured with response time [11], researchers found that font size did not influence either lexical decision time or self-paced study time, refuting the possible mediating effect of processing fluency on the font-size effect. However, it is likely that the font sizes (18 pt and 48 pt) used in previous studies may be equally easy to read. Thus, the present study asked participants to study words printed in 9 pt and 70 pt [34] font and measured self-paced study time as the indicator of processing fluency. The first aim of the present study was to examine whether processing fluency measured by study time could mediate the font-size effect.

As shown in Table 1, we find that beliefs about font size and memory have been proven to contribute to the font-size effect. Research has shown that beliefs about font size and memory exert an influence in a top-down manner [24, 11]; that is, people believe that large words will be recalled better than small words and make JOLs accordingly. In the pioneering study by Mueller et al. [11], people’s beliefs about font size and memory were measured via two approaches. Participants made GPREDs in Experiment 3, while in Experiment 4, participants made JOLs before the presentation of the words to be studied (i.e., pre-study JOLs) with information only about the font size of the words. The results demonstrated that both GPREDs and pre-study JOLs made towards large words were significantly higher than those towards small words. Combined with the significant effect of font size on JOLs, the researchers deduced that people’s beliefs about font size and memory underlie the font-size effect. However, neither the study by Mueller et al. nor other studies have linked belief judgments and item-level JOLs to examine the contribution of people’s beliefs about font size and memory to item-level JOLs. As mentioned, we reanalyzed the data of Hu et al. [24] with a mixed model that directly linked people’s beliefs about font size and memory (difference in GPREDs) to item-level JOLs. We found a significant interaction of font size and beliefs on JOLs, indicating a moderating effect of beliefs: the more strongly people believed that large words would be remembered better than small words, the higher the JOLs they would assign to large words compared with small words, a pattern similar to that demonstrated in the volume effect [9]. Thus, the present study aimed to examine whether people’s preexisting beliefs about font size and memory contribute to the font-size effect as a moderator.

In the current study, we also aimed to examine whether online generated beliefs, item-specific beliefs about fluency, could contribute to the font-size effect. According to the Metacognitive Affective Model of Self-Regulated Learning (the MASRL model) [35], when people engage in a specific task (the Task×Person level in the MASRL model), their preexisting explicit knowledge can influence self-regulation in a top-down manner. In addition, metacognitive feelings produced after involving the task can exert an influence on behavior in a bottom-up manner, in which they are produced automatically but are the objects of awareness and influence self-regulation. In this case, people are supposed to generate online knowledge or beliefs about task and cognitive processing as a mixture of the top-down and bottom-up influences exerted by preexisting knowledge and consciously experienced metacognitive feelings, respectively. Analogized to the font-size effect, it is possible that people may have a preexisting belief that larger words are easier to process (belief about font size and fluency) [11]. After actually studying a large word, however, the experienced fluency may be not as much as expected or may be greater than expected. Thus, generated knowledge indicates that the fluency of the large word is lower or higher than expected. To capture online generated item-specific beliefs about fluency, we asked participants to report the ease of reading (EOR) of each word after studying them. There are two main sources of EORs: beliefs about font size and fluency and actual experienced processing fluency. Thus, EOR is a mixture of beliefs and processing fluency. It is assumed to be a basis of JOLs and to mediate the font-size effect.

In summary, in the present study, we aimed to examine the simultaneous contribution of processing fluency and beliefs to the font-size effect. We directly measured processing fluency by self-paced study time [11, 20]. At the same time, we measured beliefs through two approaches, GPREDs [32, 30], which tap preexisting beliefs about font size and memory, and ease of learning judgments (EORs), which tap online generated item-specific beliefs about fluency. In Experiment 1, we aimed to examine whether study time and EORs could simultaneously mediate the effect of font size on JOLs. We had participants learn 9 pt and 70 pt words [34] at their own pace and make 0–100% JOLs. Immediately after making a JOL for a word, participants made an EOR for that word. We conducted a multilevel mediation analysis (level 1: items, level 2: participants) to estimate the mediating effect of study time/EORs on the font-size effect. In Experiments 2a and 2b, we aimed to examine whether preexisting beliefs about font size and memory could moderate the font-size effect [9] and, at the same time, whether item-specific beliefs about fluency and study time could mediate the font-size effect. Participants made GPREDs on the first day of the experiment, as in Mueller et al. [11] (see also Hu et al. [24]). Twenty-four hours later, participants returned to finish a study-test task. In Experiment 2a, the study-test task was the same as in Experiment 1. In Experiment 2b, JOLs and EORs were made separately; the participants made EORs after they had studied and tested all items.

## Experiment 1

Experiment 1 aimed to investigate whether processing fluency measured by self-paced study time and online generated item-specific beliefs about fluency measured by EORs could mediate the effect of font size on JOLs simultaneously.

### Participants

According to the previous studies’ effect sizes of the font-size effect (η^2^ ranging from 0.13 to 0.5 [32]), 6–24 participants are necessary to achieve a significant (α = 0.05) font-size effect at 95% power. The participants in Experiment 1 included 30 students from Beijing Normal University (9 men, 21 women). Each participant was tested individually and received 25 Renminbi (RMB; the currency unit of China) as a reward after the experiment. Written informed consent was obtained from all participants. All procedures in this experiment were approved by the Institutional Review Board of the State Key Laboratory of Cognitive Neuroscience and Learning, Beijing Normal University.

### Materials

A set of 44 Chinese 2-character words, such as 医院 (hospital), were selected from a Chinese database [36], with a word frequency of between 0.03 and 7.33 per million words. Four words were used for practice, and the remaining 40 were used for the experiment, of which 4 were used as either primary or recency buffer words and were excluded from all reported analyses.

### Procedure

The experiment consisted of three parts: the study phase, the distractor task and the free recall test. During the study phase, participants studied 40 words at their own pace. Half of the words were presented in 9 pt font and the other half in 70 pt font. The first and last two words were buffers and were presented in a fixed sequence. The remaining 36 words were presented randomly anew for each participant, with no more than 3 words presented consecutively in the same font size. Each trial began with a white blank screen for 500 ms. A word was then presented at the center of a white screen for the participants to study. If the participant thought he/she had learned the word well, then he/she hit the space key, and the word disappeared. The study time was recorded. Participants were then asked to make a judgment about the likelihood of future recall (i.e., a JOL) on a scale from 0 (cannot remember at all) to 100 (certain to remember). Immediately after the JOL, a prompt regarding “the ease of reading the word” was presented (i.e., EOR). Participants responded to the prompt on a 9-point scale, with 1 indicating difficult and 9 indicating easy. Immediately following the study list, the participants engaged in mathematics exercises for 2 min as a distractor task. Finally, the participants were asked to recall as many words as possible and typed the answers into the computer.

### Results and discussion

The means (and standard deviations) of JOLs, EORs, study time and recall performance for large and small words are presented in Table 2. Participants made higher JOLs for large words than for smaller words, *t*(29) = 3.634, *p* = 0.001, *d* = 0.664, while recall performance was not affected by font size, *t*(29) = -0.294, *p* = 0.771, *d* = 0.054, which replicated the font-size effect [32]. In addition, participants made higher EORs for large words than for smaller words, *t*(29) = 2.863, *p* = 0.008, *d* = 0.523. However, the study time was not influenced by the font size, *t*(29) = 1.338, *p* = 0.191, *d* = 0.244, refuting the hypothesized mediating effect of study time on the font-size effect.

**Table 2:** Basic descriptive statistics for Experiments 1, 2a and 2b.

	Font size		Paired t-test	
	Large	Smaller	*t*	*d*
Experiment 1				
JOL (%)	63.89(16.82)	57.45(16.76)	3.634**	0.664
EOR	6.47(1.36)	5.91(1.43)	2.863**	0.523
Study time(s)	5.52(6.80)	5.26(6.57)	1.338	0.244
Recall (%)	36.11(27.83)	36.67(28.50)	-0.294	0.054
Experiment 2a				
GPRED (%)	61.85(14.20)	50.19(12.84)	3.912**	0.714
JOL (%)	70.26(16.00)	59.27(21.09)	4.487***	0.819
EOR	7.27(1.22)	6.20(1.74)	5.037***	0.920
Study time(s)	9.40(7.32)	8.54(5.66)	1.665	0.304
Recall (%)	51.85(24.82)	54.07(25.06)	-1.046	0.191
Experiment 2b				
GPRED (%)	57.78(10.68)	44.26(11.99)	8.267***	1.509
JOL (%)	56.12(16.88)	43.06(18.83)	4.982***	0.910
EOR	6.68(1.23)	4.46(1.46)	6.023***	1.100
Recall (%)	42.04(22.54)	38.70(21.91)	1.195	0.218

**Note**. Values represent the means (and standard deviations) and the results of paired-t test for GPREDs, JOLs, EORs, study time and recall performance. GPRED = pre-study global differentiated prediction, JOL = judgment of learning, EOR = ease of learning, ST = study time.

***p < 0.001,

**p < 0.01,

*p < 0.05.

**Fig 1:**
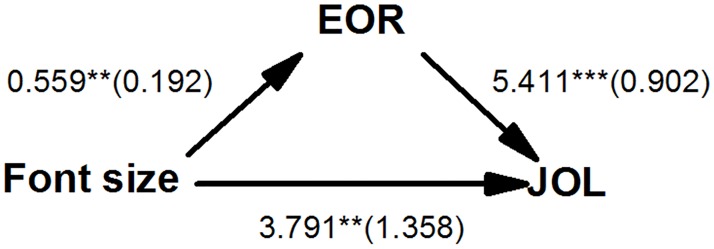
Regression coefficients (and standard errors) for the effect of font size on EORs and effects of font size and EORs on JOLs. JOL = judgment of learning, EOR = ease of reading judgment. ***p < 0.001, **p < 0.01, *p < 0.05.

To examine whether the effect of font size on JOLs was mediated by EORs, we first used MPLUS 7.11 [37] to conduct a multilevel model (level 1: items, level 2: participants) predicting JOLs [38]. EORs were regressed on font size in the first model, and JOL was regressed on font size and EORs in the second model. Fig 1 shows the coefficients. Because the direct effect of font size on EORs and the effects of font size and EORs on JOLs were all significant, EORs partially mediated the font-size effect. We further conducted a multilevel mediation analysis [39]. We coded small font size as 0 and large font size as 1. EORs were centered by grand mean. The indirect effects of font size on JOLs mediated by EORs were 3.113, 95% CI [1.159, 5.067], *p* = 0.002.

In sum, the mediation analysis revealed that online generated item-specific beliefs about fluency, measured by participants’ judgments about the ease of reading words (EORs), partially mediated the effect of font size on JOLs. However, it seems that font size did not produce a significant difference in processing fluency measured by self-paced study time. Previous studies have shown that preexisting beliefs about font size and memory are the basis of JOLs [11, 24]. In Experiment 2, we aimed to examine the simultaneous contribution of online generated item-specific beliefs about fluency and preexisting beliefs about font size and memory to the font-size effect.

## Experiment 2a

In Experiment 2a, we had participants make GPREDs on the first day, as in Hu’s Experiment 2 [24]. GPREDs of small words were subtracted from large words as “beliefs” about font size and memory. Twenty-four hours later, the participants engaged in a study-test task, which was the same as in Experiment 1, and made JOLs and EORs. Although different font sizes did not generate a difference in study time (Experiment 1), we still recorded study time in Experiment 2a to replicate it.

### Methods

The participants in Experiment 2a included 30 students (6 men, 24 women). Each participant was tested individually and received 30 RMB as a reward after the experiment.

The materials in Experiment 2a were the same as those in Experiment 1. The experiment occurred over two days and consisted of two separate tasks, the GPREDs task and the study-test task, which occurred 24 hours apart. On the first day, participants took part in the GPREDs task. They first read a description about the details of Experiment 1, except for the EORs, and observed rectangles that represented different font sizes. They were immediately asked to predict their recall performance for 9 pt and 70 pt words separately (i.e., if you took part in the experiment described above, how many 9 pt words do you think you would successfully recall? How many 70 pt words do you think you would successfully recall?) The order in which the estimates were elicited was counterbalanced across participants. Twenty-four hours after the GPREDs task, the participants returned and finished the study-test task. The procedure of this task was the same as in Experiment 1.

### Results and discussion

The means (and standard deviations) of GPREDs, JOLs, EORs, study time and recall performance for large and small words are presented in Table 2. Participants made higher JOLs for large words than for small words, *t*(29) = 4.487, *p* < 0.001, *d* = 0.819; however, recall performance was not affected by font size, *t*(29) = -1.046, *p* = 0.304, *d* = 0.191. Participants’ GPREDs of large words were significantly higher than those of small words, *t*(29) = 3.912, *p* = 0.001, *d* = 0.714, demonstrating their preexisting beliefs about font size and memory. In addition, participants again made higher EORs for large words than for small words, *t*(29) = 5.037, *p* < 0.001, *d* = 0.920, whereas font size did not produce a difference in study time, *t*(29) = 1.665, *p* = 0.107, *d* = 0.304.

We then conducted a multilevel model to examine whether the font-size effect was moderated by beliefs about font size and memory and mediated by item-specific beliefs about fluency simultaneously. We coded small size as 0 and large size as 1. EORs were centered by the grand mean. GPREDs of small words were subtracted from GPREDs of large words as “beliefs” about font size and memory. The results demonstrated that the effect of font size on JOLs was mediated by EORs. The indirect effect was 6.522, *SE* = 1.572, 95% CI [3.441, 9.603], *p* < 0.001. Moreover, the effect of font size on JOLs was significantly moderated by beliefs about font size and memory simultaneously, β = 0.273, *SE* = 0.127, 95% CI [0.024, 0.522], *p* = 0.032. The significant moderating effect indicated that the stronger the belief that large words would be recalled better, the more likely it was that higher JOLs would be given to large words rather than to smaller words.

Experiment 2a successfully showed that the font-size effect was mediated by participants’ online generated beliefs about fluency (EORs) and simultaneously moderated by their preexisting beliefs about font size and memory (difference in GPREDs). It is worth noting that the participants made EORs immediately after JOLs, which might have led to the agreement of these two judgments. That is, participants might have made EORs according to the preceding JOLs: the higher the JOLs, the higher the EORs. Importantly, this consistency might result in the significant indirect effect of EORs we demonstrated previously. To rule out this possible side effect, we isolated the two judgments in Experiment 2b. In the second day’s study-test task in Experiment 2b, the participants first studied and made JOLs for different font-size words and finished a free recall task. Finally, they made EORs for the words studied previously one by one.

## Experiment 2b

Experiment 2b aimed to replicate the concurrent mediating effect of EORs and the moderating effect of beliefs about font size and memory in the font-size effect while eliminating the possible consistency between JOLs and EORs deriving from the experimental manipulation. Because study time was found to have no impact on the font-size effect (Experiment 1 and Experiment 2a), we fixed the study time in Experiment 2b.

### Methods

The participants in Experiment 2b included 30 students from Beijing Normal University (6 men, 24 women).

The materials in Experiment 2b were the same as those in Experiment 2a. The procedure in Experiment 2b was also the same as that in Experiment 2a, except EORs were not given immediately after JOLs in the study-test task. Rather, EORs were given after participants had studied and been tested on all words. All studied words were presented one by one, and participants made EORs at their own pace. Another difference in the manipulation was that we fixed the study time at 5 s per item.

### Results and discussion

The means (and standard deviations) of GPREDs, JOLs, EORs and recall performance for large and small words are presented in Table 2. Font size significantly influenced JOLs, demonstrating higher JOLs for large words than for small words, *t*(29) = 4.982, *p* < 0.001, *d* = 0.910, but did not influence recall performance, *t*(29) = 1.195, *p* = 0.242, *d* = 0.218. Participants made higher GPREDs towards large words compared with small words, *t*(29) = 8.267, *p* < 0.001, *d* = 1.509. Participants also made higher EORs for large words than for small words, *t*(29) = 6.023, *p* < 0.001, *d* = 1.100.

We then conducted the same multilevel model as in Experiment 2a. We found that the effect of font size on JOLs was mediated by EORs. The indirect effect was 5.729, *SE* = 1.675, 95% CI [2.446, 9.012], *p* = 0.001. At the same time, the effect of font size on JOLs was significantly moderated by beliefs about font size and memory, β = 0.594, *SE* = 0.208, 95% CI [0.187, 1.002], *p* = 0.004.

In summary, Experiment 2b successfully replicated the concurrent mediating effect of EORs and the moderating effect of beliefs about font size and memory after excluding the effect of an agreement of EORs and JOLs, which indicated that the mediating effect of online generated item-specific beliefs about fluency in the font-size effect was not due to the side effect of experimental manipulation.

## General discussion

The present study aimed to examine whether and how processing fluency (study time), preexisting beliefs about font size and memory (difference in GPREDs), and online generated item-specific beliefs about fluency (EORs) contribute to the font-size effect during the same study phase simultaneously. In Experiment 1, participants studied 9 pt and 70 pt words under a self-paced study condition and made JOLs and EORs. The findings suggested that participants gave higher EORs to large words than to smaller words, and EORs significantly mediated the effect of font size on JOLs, while no difference occurred in study time. In Experiments 2a and 2b, participants engaged in a GPREDs task on the first day and a study-test task identical to the task in Experiment 1 24 hours later. We found that EORs significantly mediated the font-size effect; at the same time, GPREDs moderated the font-size effect. Moreover, the pattern of the results was maintained when we excluded the possible agreement between JOLs and EORs stemming from experimental manipulation (Experiment 2b). In summary, the present study suggests the important role of beliefs in the font-size effect.

In Experiments 1 and 2a, the nonsignificant effect of font size on self-paced study time replicated the results of Mueller et al. [11], although we utilized a pair of font sizes with larger visual differences, suggesting that font size may not produce differences in processing fluency measured by study time. Recently, a study by Yang et al. [22] found that identification time in a continuous identification task mediated the effect of font size on JOLs. Thus, it is possible that study time is not a sensitive measure of fluency. As Yang et al. proposed in their study, factors other than fluency might influence people’s study time, such as beliefs that large words are more important than small words, leading to a greater allocation of study time to large words. We conjecture that the difference in processing time derived from different visual sizes (0.25 s in Experiment 1 of Yang et al.) can be covered by a much longer conceptual processing time. In the self-paced study phase, to recall more words in the future test, participants spent considerable time on encoding, such as conceptual elaboration and mental imagery. The time consumed constitutes the majority of study time, leading to the insensitivity of study time to fluency produced by visual size. Thus, in this study, although study time was not affected by font size, we cannot conclude that fluency did not contribute to the font-size effect because study time may not be a good indicator of fluency.

Experiments 2a and 2b linked people’s preexisting beliefs about font size and memory with item-level JOLs, identifying a moderating effect of preexisting beliefs on the font-size effect, which has also been demonstrated with regard to the volume effect [29]. The moderating effect of preexisting beliefs demonstrates that the stronger the belief that large words will be recalled better than smaller words, the more likely it is that higher JOLs will be given to large words than to smaller words. Other studies have also partially supported the moderating effect of preexisting beliefs on JOLs [24, 31]. The study by Hu et al. [24] revealed a positive prediction of preexisting beliefs about font size and memory to JOLs at the participant level. Mueller and Dunlosky [31] (Experiment 5 and its replication and extension experiment) found that participants who believed that blue (vs. green) was easier to process made higher JOLs for blue words, while participants who reported that neither color was easier to process did not make different JOLs for blue and green words. All of these studies demonstrate that people’s preexisting beliefs influence JOLs in a top-down manner [35].

The present study demonstrated that online generated item-specific beliefs about fluency, measured by EORs, mediated the font-size effect. According to the MASRL model [35], EORs are supposed to be a mixture of the top-down influence of preexisting beliefs about font size and fluency and the bottom-up influence of the consciously experienced processing fluency. To examine the influence of beliefs about font size and fluency on EORs, we conducted an extension experiment as an observer group of Experiment 2a to construct a learner-observer paradigm as in Experiment 1 of Hu et al.[24]. In the extension study, 30 observers were yoked with the 30 learners in Experiment 2a. The observers completed an online questionnaire. They were told about Experiment 2a and presented with 2 rectangles illustrating the size of the two-character Chinese words in 9 pt and 70 pt. They were presented with words in the same size along with the word “large” or “small”, indicating the font size of the specific word the yoked learner studied, and were asked to predict the EORs the learner made. Because visual differences in font size were removed, the effect of font size on EORs could only be based on beliefs about font size and fluency. One participant was removed because her response time (118.55 min) was far beyond the average response time (Mean = 9.12, SD = 20.80). We found that observers predicted higher EORs towards large words (Mean = 6.29, SD = 1.05) compared with small words (Mean = 4.41, SD = 1.38), *t*(28) = 5.426, *p* < 0.001, *d* = 1.008, demonstrating beliefs about font size and fluency. In addition, we found that the difference in EORs between large and small words was larger in observers (Mean = 1.87, SD = 1.86) than in learners (Mean = 1.07, SD = 1.17), *t*(57) = 1.986, *p* = 0.052, *d* = 0.515. We speculate that the influence of actual experienced fluency on learners’ EORs might be a source of this difference. To further examine the contribution of processing fluency to EORs, we conducted a multilevel model predicting EORs with study time. The results demonstrated that EORs could not be predicted by study time, β = 0.009, *SE* = 0.263, 95% CI [-0.505, 0.524], *p* = 0.972 in Experiment 1 and β = -0.001, *SE* = 0.005, 95% CI [-0.011, 0.009], *p* = 0.837 in Experiment 2a. Despite the fact that study time cannot predict EORs, we cannot conclude that EORs did not derive from people’s perceived feeling of fluency. As mentioned previously, study time may not be a sensitive indicator of fluency. In brief, we demonstrated that EORs were affected by beliefs about font size and fluency along with a possible influence of processing fluency (although we did not find a direct effect of study time on EORs). Future studies can directly measure people’s beliefs about font size and fluency and utilize a more sensitive indicator of fluency to demonstrate their relationship with EORs.

Our results show that people have beliefs about font size and fluency, providing evidence supporting the idea of Mueller and Dunlosky [31]. Mueller and Dunlosky proposed that when people are asked to make JOLs, they will search for cues and retrieve a prior belief or develop a belief about the cues online to reduce their uncertainty. In the context of the font-size effect, people may generate a belief that larger words are easier to process (belief about font size and fluency) and a belief that easier processing leads to better recall performance (belief about fluency and memory). These authors demonstrated the influence of beliefs about fluency and memory on JOLs, though not in the context of the font-size effect. The present study further demonstrated that people do have beliefs about font size and fluency. Moreover, we found that these beliefs (likely along with consciously experienced fluency) may exert an influence on online generated item-specific beliefs about fluency, which mediate the font-size effect at the item level. Combined with the proven influence of beliefs about fluency and memory, the results suggest a possible multiple mediating effect of beliefs underlying the font-size effect. That is, font size may give rise to both beliefs about font size and fluency and processing fluency. Item-specific beliefs about fluency as a mixture further contribute to JOLs through beliefs about fluency and memory.

Although the present study did not provide as much evidence supporting EORs as the mixture of preexisting beliefs and actual processing experience, previous studies have revealed that participants’ naive theories or beliefs interact with task-level experience when people make judgments [40–42]. A representative finding was that people’s beliefs about intelligence may influence how people explain the effort invested in encoding and further influence JOLs [40]. People who believed that intelligence was fixed tended to interpret effortful encoding as a result of limited ability and gave higher JOLs to high-fluency items, whereas people who believed that intelligence developed incrementally tended to interpret effort as a key to performance and gave higher JOLs to low-fluency items. Another study directly manipulated people’s beliefs about memory to determine its influence on inferences from retrieval fluency [42]. The results demonstrated that when participants were required to recall 12 childhood events (a difficult task), participants rated their childhood as happier when they were led to believe that pleasant rather than unpleasant periods of life are difficult to recall. In contrast, when the task was easy, recalling 4 childhood events, participants rated their childhood as less happy when they were led to believe that unpleasant rather than pleasant periods of life are difficult to recall. We suggest that future studies pay attention to the possible interaction of beliefs and processing experience and its influence on JOLs.

## Supporting information

S1 Table Basic descriptive statistics (Table A) and results of multilevel model predicting JOLs (Table B) of Experiment 2 of Hu et al. (DOCX)

S1 Data Data collected in Experiment 1. (CSV)

S2 Data Data collected in Experiment 2a. (CSV)

S3 Data Data collected in Experiment 2b. (CSV)
