# Supplementary material for: How font size affects judgments of learning: Simultaneous mediating effect of item-specific beliefs about fluency and moderating effect of beliefs about font size and memory
Source: PLoS One. 2018 Jul 20;13(7):e0200888. doi: 10.1371/journal.pone.0200888 (PMC6054382; doi:10.1371/journal.pone.0200888)
Supplement: S1 Table — (DOCX) [file pone.0200888.s001.docx]

**Table A. Basic Descriptive Statistics for Experiment 2 of Hu et al.**

|  | Font size | |
| --- | --- | --- |
|  | Large | Smaller |
| GPRED (%) | 58.00(11.73) | 42.80(13.08) |
| JOL (%) | 55.46(13.01) | 47(14.10) |
| Recall (%) | 47(14.10) | 47(14.10) |

**Note.** Values represent the means (and standard deviations) for GPREDs, JOLs and recall performance. GPRED = pre-study global differentiated prediction, JOL = judgment of learning.

**Table B. Results of Multilevel Model Predicting JOLs of Experiment 2 of Hu et al.**

| Predictor variable | β | *SE* | 95% CI | *p* |
| --- | --- | --- | --- | --- |
| Font size | 3.114 | 1.452 | [0.268, 5.959] | 0.032 |
| Beliefs | -8.337 | 22.112 | [-51.676, 35.002] | 0.706 |
| Font size × beliefs | 0.331 | 0.079 | [0.176, 0.486] | <0.001 |
